# Refurbishing Pacemakers: A Viable Approach

**Published:** 2004-01-01

**Authors:** R Anilkumar, J Balachander

**Affiliations:** Department of Cardiology, JIPMER, Pondicherry-605006, India

Cardiologists implant permanent pacemakers widely for indications like sick sinus syndrome and complete heart block. The guidelines for such implantations are well established [1]. However, in developing countries like India, all patients who need pacemakers do not receive them because of financial constraints. Even when such patients get a pacemaker, it is often a more affordable VVI pacemaker rather than the costly DDD pacemaker. The lack of a health insurance scheme and improper social support programs prevent the more widespread implantation of appropriate pacemakers.

However, in the developed countries and in affluent pockets of developing countries like India, the pacemaker implantation rates are quite high.  Often permanent pacemakers are implanted in the very old and people with predicted brief longevities, due to medico-legal and other social reasons. There are quite a few instances when pacemakers are explanted within a year or even within a few months. This is often due to the unfortunate death of the patient due to unrelated causes. Such pacemakers have battery lives, which are near normal. These can be explanted from the dead patient after taking consent from the relatives and “refurbished” for use in another needy patient. Refurbishing involves proper re-sterilization, checking of battery life, pacing mode and other parameters and re-labelling with the current parameters including predicted battery life. These refurbished pacemakers are a suitable alternative for the financially ‡no option’ group of patients who otherwise would not afford a pacemaker. These can last nearly as long as the original pacemakers. Even pulse generators whose shelf lives have expired can also be resterilised and used gainfully for the economically deprived.

Nampoothiri et al, in this issue of the journal, have dwelt upon this option [2]. They have explanted DDD pacemakers from patients who have expired and implanted as VDD pacemaker in a new patient with a VDD lead.  The authors have stressed on the use of VDD Medtronic lead being more economical than two Medtronic leads. But two leads, one atrial and one ventricular, available from an Indian company, can be even cheaper than the VDD lead. The article however clearly shows the effective use of refurbished pacemakers.

Refurbishment of pacemakers requires the active co-operation of medical and cardiology units and the emergency services department of various hospitals.  The pacemakers need to be explanted from patients who expired, after consent from the relatives. Then, these pacemakers need to be checked for their integrity and battery life, sterilized re-labeled and kept ready for use in the needy patient.

STIMUBANK is one such organization in Nancy, France, which collects pacemakers that are prematurely removed, or shelf expired pacemakers from various parts of France. Pulse generators having a potential life of more than four years, i.e. the guarantee period of pacemakers in France, are shipped to needy centers outside France. Legislation in France does not permit the re use of implanted devices.

JIPMER, Pondicherry, has been the largest beneficiary of STIMUBANK and has a large experience of implanting refurbished pacemakers [3]. This center has followed up over five hundred patients with refurbished pacemakers over the last twenty years. The longevity, performance and complications compare reasonably well with the newly implanted pulse generators. There are smaller series of refurbished pacemakers from other centers as well [4,5]

Specific consent has to be sought from the patient and his relatives regarding the implantation of a refurbished pacemaker. The parameters of the pacemaker also have to be checked thoroughly before implant. This requires that programmers of all makes of refurbished pacemakers be available at the center implanting such pacemakers. Inadvertently, implantation of a pacemaker programmed for bipolar pacing with a unipolar pacing lead can result in failure of pacing. Hence it is very essential that proper assessment of pacing parameters be done very judiciously and adequate battery life ensured before embarking on implantation of a refurbished pacemaker.

A good number of pacemakers with reasonable battery lives can be salvaged with some planning and foresight. This could even be extended to some defibrillators and triple chamber pacemakers whose batteries   outlive the patients in whom they are implanted. A central registry or bank, akin to the STIMUBANK, can be formed in India where explanted pacemakers from anywhere in the country can be sent. This organization can refurbish the pacemakers at a very minimal cost and supply, on request, to the needy patients. Thus, with just the cost of one or two pacemaker leads, a single or the costlier dual chamber pacemaker, as indicated, can benefit the economically deprived needy patient. This option needs to be seriously considered.
